# Second-line Treatment of Non-Small Cell Lung Cancer: Focus on the Clinical Development of Dacomitinib

**DOI:** 10.3389/fmed.2017.00036

**Published:** 2017-04-05

**Authors:** Jon Zugazagoitia, Asunción Díaz, Elisabeth Jimenez, Juan Antonio Nuñez, Lara Iglesias, Santiago Ponce-Aix, Luis Paz-Ares

**Affiliations:** ^1^Medical Oncology Department, Hospital Universitario 12 de Octubre, Instituto de Investigación i+12, Madrid, Spain; ^2^Complutense University, Madrid, Spain

**Keywords:** non-small cell lung cancer, second-line treatment, EGFR mutations, second-generation EGFR tyrosine-kinase inhibitors, dacomitinib, acquired resistance

## Abstract

Dacomitinib is a second-generation, irreversible, covalent pan-HER tyrosine-kinase inhibitor (TKI). It showed potent EGFR signaling inhibition in experimental models, including first-generation TKI-resistant non-small cell lung cancer (NSCLC) cell lines. This preclinical efficacy did not translate into clinically meaningful treatment benefits for advanced, pretreated, molecularly unselected NSCLC patients enrolled in two parallel phase III trials. Dacomitinib and erlotinib showed overlapping efficacy data in chemotherapy-pretreated *EGFR* wild-type (WT) patients in the ARCHER 1009 trial. Similarly, it failed to demonstrate any survival benefits as compared to placebo in *EGFR* WT subsets progressing on chemotherapy and at least one previous first-generation TKI (erlotinib or gefitinib) in the BR.26 trial. In the case of *EGFR*-mutant NSCLCs, a pooled analysis of the ARCHER 1009 and ARCHER 1028 trials comparing the efficacy of dacomitinib vs. erlotinib in chemotherapy-pretreated, EGFR TKI-naïve patients showed a trend to a longer progression-free survival (PFS) and overall survival in favor of dacomitinib that did not reach statistical significance, with a higher rate of treatment related adverse events (mainly skin rash, paronychia, and gastrointestinal toxicities). On the other hand, the clinical activity in patients with *EGFR*-mutant NSCLCs with acquired TKI resistance that were included in phase II/III trials was equally poor (response rate <10%; PFS 3–4 months). Therefore, with the results of the ARCHER 1050 trial (NCT01774721) still pending, the current clinical development of dacomitinib is largely focused on *EGFR*-mutant, TKI-naïve patients. Here, we review the most relevant clinical data of dacomitinib in advanced NSCLC. We discuss the potential role of dacomitinib in pretreated *EGFR* WT and *EGFR*-mutant (TKI-naïve and TKI-resistant) patients. Finally, we briefly comment the available clinical data of dacomitinib in HER2-mutant NSCLC patients.

## Introduction

Second-line treatment options for advanced non-small cell lung cancer (NSCLC) patients have substantially expanded in the past few years. Docetaxel- or pemetrexed-based chemotherapy and erlotinib were the only three drugs approved in our setting until year 2014, achieving an approximate 8–10% of response rates (RRs), median 4 months of progression-free survival (PFS) and 8–10 months of overall survival (OS) (1). Recently, antiangiogenics [ramucirumab (2), nintedanib (3), and bevacizumab (4)] and particularly PD-1/PD-L1 inhibitors [nivolumab (5, 6), pembrolizumab (7), and atezolizumab (8)] have shown to prolong survival in pretreated patients, transforming the standardization of second-line NSCLC treatment.

In the absence of significant differences in terms of efficacy, the choice between pemetrexed- or docetaxel-based second-line chemotherapy is largely driven by three factors: histology, as pemetrexed is restricted to non-squamous tumors, type of platinum doublet used during first-line treatment, with pemetrexed being increasingly incorporated into the first-line or maintenance treatments, and differences in toxicity profiles. On the other hand, when deciding between chemotherapy and erlotinib, apart from clinical factors, EGFR mutation status is the main biomarker that determines treatment selection.

The IPASS trial definitely demonstrated that the clinical activity of EGFR tyrosine-kinase inhibitors (TKIs) in treatment-naïve patients was restricted to those with *EGFR*-mutant tumors (*EGFR*-sensitizing mutations). As the clinical activity of EGFR TKIs in TKI-naïve, *EGFR*-mutant tumors is comparable between treatment-naïve or platinum-pretreated patients (9), first- or second-generation EGFR TKIs are the preferred treatment options in patients with *EGFR*-mutant tumors. On the contrary, in patients with *EGFR* wild-type (WT) cancers, RRs and survival were significantly lower with gefitinib- compared to platinum-based chemotherapy in the IPASS study (10). However, whether this was also true in the second-line setting, a clinical context in which the efficacy of docetaxel- or pemetrexed-based chemotherapy hardly reaches 10% of RRs, has been a matter of extensive debate in the past few years. Some molecularly unselected randomized trials, initiated at a time where no definitive predictive biomarkers for the benefit or EGFR TKIs were discovered yet, initially suggested similar efficacy outcomes between erlotinib and second-line chemotherapy (11–13). More recent data, including molecularly selected or molecularly stratified randomized trials and large meta-analysis, have confirmed that second-line chemotherapy is superior to EGFR TKIs in patients with *EGFR* WT tumors, at least in terms of RRs and PFS. OS differences did not reach statistical significance (14–16).

In this therapeutic scenario, and considering that EGFR pathway activation might hypothetically contribute to cancer progression even in tumors with no *EGFR* activating mutations (17), to investigate if a more potent pan-HER inhibition with dacomitinib would add any clinical benefit seemed a rational approach, either from a biological or a clinical perspective. In addition, as the majority of patients with *EGFR*-mutant tumors treated with first-generation EGFR TKIs develop acquired resistance by ERBB-dependent mechanisms (18), and considering that dacomitinib showed activity in gefitinib-resistant preclinical lung cancer models (19), it was also rational to test its clinical activity in patients with *EGFR*-mutant, TKI-resistant cancers. Herein, we will succinctly discuss the potential role of second-line dacomitinib in *EGFR* WT and *EGFR*-mutant NSCLC.

## Dacomitinib: Preclinical and Early Clinical Data in NSCLC

Dacomitinib is a second-generation, irreversible, covalent-binding pan-HER TKI. As compared to first-generation EGFR TKIs, it has comparable inhibitory activity against the WT EGFR kinase *in vitro*. However, dacomitinib is more potent than gefitinib against cell lines harboring common *EGFR*-sensitizing mutations (del19, L858R). Moreover, it has inhibitory activity against gefitinib-resistant exon 20 insertions and acquired resistance exon 20 T790M mutations in preclinical lung cancer models. Unlike gefitinib or other first-generation TKIs, dacomitinib, as a pan-ERBB inhibitor, also inhibits the activity of both WT and mutant HER2 kinase (19, 20).

Three phase I trials, conducted both in Western and Asian patients, established that the maximum tolerated dose of dacomitinib was 45 mg daily, and this dose level was selected for further clinical evaluation. The most frequent dose-limiting drug-related adverse events were skin and gastrointestinal toxicities (21–23). The three trials consistently demonstrated that plasma concentrations and other pharmacokinetic parameters proportionally increased with increasing doses of oral dacomitinib (21–23), with no apparent food effect (21). Dacomitinib’s half-life was estimated at 59–85 h in the phase I trial conducted in the United States (21). A modest preliminary clinical activity was observed in small cohorts of NSCLC patients previously treated with first-generation EGFR TKIs and/or chemotherapy. No objective responses were seen in EGFR TKI-resistant patients whose tumors harbored *EGFR* T790M mutations (21–23).

## Dacomitinib for Pretreated NSCLC Patients

### Clinical Data in *EGFR* WT or NSCLCs Unselected by *EGFR* Status

The clinical activity of dacomitinib in pretreated NSCLC patients has been evaluated in four clinical trials (24–27). They are mostly molecularly unselected trials and, consequently, the vast majority of the patients included had *EGFR* WT tumors. An overview of the four clinical trials and the efficacy data in the overall study population are summarized in Table 1.

**Table 1:** **Clinical studies and efficacy data of dacomitinib in pretreated, advanced NSCLC patients**.

Study	Phase	Clinical context	Molecular eligibility	*N* overall	*N* EGFR mutant	*N* EGFR WT	Response rates	PFS	OS
ARCHER 1002 (25)	Single arm phase II	Pretreated patients who failed at least one chemotherapy regimen, but no more than two, and erlotinib	KRAS WT^a^	66	26	23	5.2%	12 weeks	37 weeks
ARCHER 1028 (24)	Randomized phase II	Pretreated, TKI-naive patients who failed one or two chemotherapy regimens	Unselected	188	30	129	Dacomitinib: 17%	Dacomitinib: 2.86 months	Dacomitinib: 9.53 months
							Erlotinib: 5.3%	Erlotinib: 1.91 months	Erlotinib: 7.44 months
							*p* = 0.01	HR 0.66 (CI 95% 0.47–0.91; *p* = 0.01)	HR 0.80 (CI 95% 0.56–1.10; *p* = 0.20)
BR.26 (27)	Phase III	Pretreated patients who failed up to three chemotherapy regimen and erlotinib/gefitinib	Unselected	720	182	349	Dacomitinib: 7%	Dacomitinib: 2.66 months	Dacomitinib: 6.83 months
							Placebo: 1%	Placebo: 1.38 months	Placebo: 6.31 months
							*p* = 0.001	HR 0.66 (CI 95% 0.55–0.79)	HR 1.00 (CI 95% 0.83–1.21)
ARCHER 1009 (26)	Phase III	Pretreated, TKI-naive patients who failed one or two chemotherapy regimens	Unselected	878	91	662	Dacomitinib: 17%	Dacomitinib: 2.6 months	Dacomitinib: 7.9 months
							Erlotinib: 5.3%	Erlotinib: 2.6 months	Erlotinib: 8.4 months
							*p* = 0.01	HR 0.94 (CI 95% 0.80–1.10; *p* = 0.22)	HR 1.07 (CI 95% 0.91–1.27; *p* = 0.81)

*^a^Patients with EGFR-mutant tumors were assumed to be KRAS WT based on mutual exclusivity*.

*PFS, progression-free survival; OS, overall survival; TKI, tyrosine-kinase inhibitor; NSCLC, non-small cell lung cancer; WT, wild-type*.

Two phase II trials initially suggested some degree of clinical activity in pretreated NSCLC patients. The ARCHER 1002 trial was a single-arm study that tested the activity of dacomitinib in patients that were refractory to one or two lines of chemotherapy and erlotinib. On the basis that *KRAS* mutant cell lines were primarily resistant to first- or second-generation EGFR TKIs, this study was enriched with patients with *KRAS* WT tumors. The trial failed to meet its primary end point, as dacomitinib yielded a disappointing 5.2 and 4.8% of RRs in the overall and adenocarcinoma subsets, respectively. Patients with *EGFR* WT/*KRAS* WT tumors included in this trial had comparable RRs (5%), PFS (8 weeks), and OS (26 weeks) to those of the overall study population (25) (Table 1). The second phase II trial (ARCHER 1028) compared the activity of dacomitinib and erlotinib in molecularly unselected patients progressing on one or two prior chemotherapy regimens. In this case, the trial met its primary endpoint, showing a statistically significant increase in PFS (2.86 vs. 1.91 months, HR 0.66, CI 95% 0.47–0.91) in favor of dacomitinib in the overall study population. Objective responses were also higher in dacomitinib treated patients (17 vs. 5.3%, *p* = 0.01). However, no differences in OS were noted (HR 0.80, CI 95% 0.56–1.10, *p* = 0.20) (Table 1). Comparable degree of PFS increment to the overall population was observed in *EGFR* WT NSCLCs (HR 0.70, CI 95% 0.47–1.05) and *EGFR* WT/*KRAS* WT NSCLCs (HR 0.61, CI 95% 0.37–0.99). Dacomitinib did not improve OS compared to erlotinib in patients with *EGFR* WT cancers (24).

This modest clinical activity served as the basis to launch two subsequent randomized phase III trials in similar therapeu-tic scenarios to their respective phase II trials. Unfortunately, both phase III studies were negative. First, in the BR.26 trial, whereas dacomitinib statistically significantly improved RRs (7 vs. 1%, *p* = 0.001) and PFS (2.66 vs. 1.38 months, HR 0.66 CI 95% 0.55–0.79) compared to placebo in patients progressing on chemotherapy and EGFR TKIs, it failed to demonstrate improved OS (primary end point; HR 1.00) (Table 1). Similarly, no trend for a clinically meaningful incremental efficacy was observed in patients with *EGFR* WT tumors or patients with both *EGFR* and *KRAS* WT NSCLCs compared to the overall patient population (27). And finally, Dacomitinib failed to improve the efficacy of erlotinib (control arm) in second- or third-line settings (ARCHER 1009), either in the overall population (Table 1) or in patients with *EGFR* WT tumors. In the latter subgroup, dacomitinib had overlapping objective RRs, PFS (1.9 vs. 1.9 months; HR 0.94, CI 95% 0.79–1.13), and OS (6.8 vs. 7.6 months; HR 1.07, CI 95% 0.90–1.29) compared to erlotinib. Results were almost identical for patients with either *KRAS* or *EGFR* WT NSCLCs (26).

### Clinical Data in *EGFR*-Mutant, TKI-Naïve NSCLCs

In the particular case of pretreated, TKI-naïve subsets, a pooled analysis of the ARCHER 1009 and ARCHER 1028 trials comparing the efficacy of dacomitinib vs. erlotinib showed a comparable median PFS (14.6 vs. 9.6 months, respectively; HR 0.71, *p* = 0.14) and OS (26.6 vs. 23.2 months, respectively; HR 0.73, *p* = 0.26) outcomes that somehow favored dacomitinib (28) (Table 2). Both ARCHER 1028 and ARCHER 1009 trials showed that on target adverse events related to the inhibition of *EGFR* WT in normal tissues were significantly increased with dacomitinib compared to erlotinib, mainly skin rash, paronychia, and gastrointestinal toxicities (24, 26). These data are in line with the recently published LUX-Lung 7 trial, where afatinib significantly delayed PFS and the emergence of EGFR TKI resistance, albeit with a higher incidence of treatment related adverse events (29).

**Table 2:** **Clinical data of dacomitinib in EGFR-mutant NSCLCs**.

Study	Phase	Clinical context	No. of patients with EGFR-mutant tumors (sensitizing mutations)	Response rates (%)	PFS	OS
A7471017 (30)	II	Treatment naive	45	76	18.2 months	–
Pooled analysis ARCHER 1009 and ARCHER 1028 (28)	II and III	Chemotherapy-pretreated, TKI naive	101	67.9	14.6 months	26.6 months
ARCHER 1002 (25)	II	TKI resistant	24	8	18 weeks	56 weeks
BR.26 (27)	III	TKI resistant	114	–	3.52 months	7.23 months

*PFS, progression-free survival; OS, overall survival; TKI, tyrosine-kinase inhibitor; NSCLCs, non-small cell lung cancers*.

### Clinical Data in *EGFR*-Mutant, TKI-Pretreated NSCLCs

In the context of EGFR TKI acquired resistance, the clinical efficacy of dacomitinib in patients with *EGFR*-mutant lung cancers progressing on first-generation EGFR TKIs that were included in these trials was disappointingly low, with an overall RR of about 8% (Table 2). No objective responses were reported among patients whose tumors harbored the secondary acquired resistance *EGFR* T790M mutation. In general, the PFS and OS data did not differ to those of the unselected patient population either (25, 27).

### Clinical Data in *HER2*-Mutant, TKI-Naïve NSCLCs

In the largest prospective phase II study conducted to date in patients with *HER2*-mutant or *HER2*-amplified tumors (*n* = 30; 83% had received at least one line of previous chemotherapy), dacomitinib showed only modest efficacy, with an objective RR of 12%, 3 months of median PFS, and 9 months of median OS. No responses were seen in patients with tumors harboring the most common *HER2* activating mutation (c. 2324_2325ins12) (31). Intriguingly, tumors with this genotype did respond to afatinib in other series (32). No responses were seen either in patients with *HER2*-amplified cancers (*n* = 4) (31). More studies are needed in order to determine which molecular contextures (i.e., possible coexistence with *HER2* amplification) and what specific *HER2* genotypes are true predictive targets for the benefit of dacomitinib.

## Conclusion and Future Perspectives

Dacomitinib has failed to improve overall outcomes in pretreated NSCLC patients. An irreversible pan-HER inhibition is not superior to erlotinib in patients with no *EGFR*-sensitizing mutations and does not prolong OS compared to placebo in heavily pretreated patients either. Also, dacomitinib does not overcome *EGFR* T790M-mediated acquired resistance in *EGFR*-mutant NSCLCs at tolerable doses in humans. In non-T790M-mediated resistance, in which functional activation of HER pathway or acquired *HER2* activating mutations have been described in some cases (18, 33), no reliable clinical data are available, but a robust activity in this clinical setting seems unlikely. With these clinical data, together with recent regulatory approvals of third-generation, *EGFR*-mutant selective TKIs (e.g., osimertinib) with potent activity against the T790M mutation (34), current development of dacomitinib is focused to TKI treatment-naïve, molecularly selected patients with *EGFR*-mutant and *HER2*-mutant lung cancers. In a small phase II trial including a total of 45 treatment-naïve patients with tumors harboring common *EGFR*-sensitizing mutations, dacomitinib achieved an overall RR of 75.6% and a median PFS of 18.2 months (30).

In this regard, whether second-generation EGFR TKIs in TKI-naïve patients are superior to first-generation TKIs in EGFR-mutant NSCLCs is not fully answered to date. In the LUX-Lung 7 trial, afatinib significantly increased RRs (70 vs. 56%; *p* = 0.0083), median PFS (11 vs. 10.9 months; HR 0.73, CI 95% 0.57–0.95; *p* = 0.0195), and median time to treatment failure (13.7 vs. 11.5 months; HR 0.73, CI 95% 0.58–0.92; *p* = 0.0073) over gefinitib. However, there were no OS differences among treatment arms in this phase IIb trial (*n* = 319). Pre-specified subgroup analysis according to mutation type (exon 19 deletions vs. L858R mutations) did no show significant differences in OS either. Overall, treatment-related adverse events (mainly skin rash and diarrhea) and serious adverse events were more common with afatinib (33). Therefore, this trial suggests that the emergence of acquired resistance might be delayed with second-generation compared to first-generation TKIs, but whether these modest differences are clinically relevant for patients is arguable for many physicians. The ARCHER 1050 trial (NCT01774721) comparing first-line dacomitinb vs. gefitinib has recently completed accrual and will hopefully give a definitive answer in this regard, establishing the true role of front-line dacomitinib in *EGFR*-mutant NSCLCs.

## Author Contributions

All authors contributed equally to this work.

## Conflict of Interest Statement

LP-A has received advisory honoraria from Roche, Lilly, MSD, Novartis, BMS, Clovis, Amgen, Boehringer Ing., Astra Zeneca, and Pfizer, not related to this work. The other authors declare that the research was conducted in the absence of any commercial or financial relationships that could be construed as a potential conflict of interest.
